# Supplementary material for: Monitoring cytosolic H2O2 fluctuations arising from altered plasma membrane gradients or from mitochondrial activity
Source: Nat Commun. 2019 Oct 4;10:4526. doi: 10.1038/s41467-019-12475-0 (PMC6778086; doi:10.1038/s41467-019-12475-0)
Supplement: Supplementary file 1 — Supplementary Information [file 41467_2019_12475_MOESM1_ESM.pdf]

SUPPLEMENTARY INFORMATION

Monitoring cytosolic H_2O_2 fluctuations arising from altered plasma membrane gradients or from mitochondrial activity

Carmona et al.

Includes:

9 supplementary figures

1 supplementary Table

SUPPLEMENTARY FIGURES

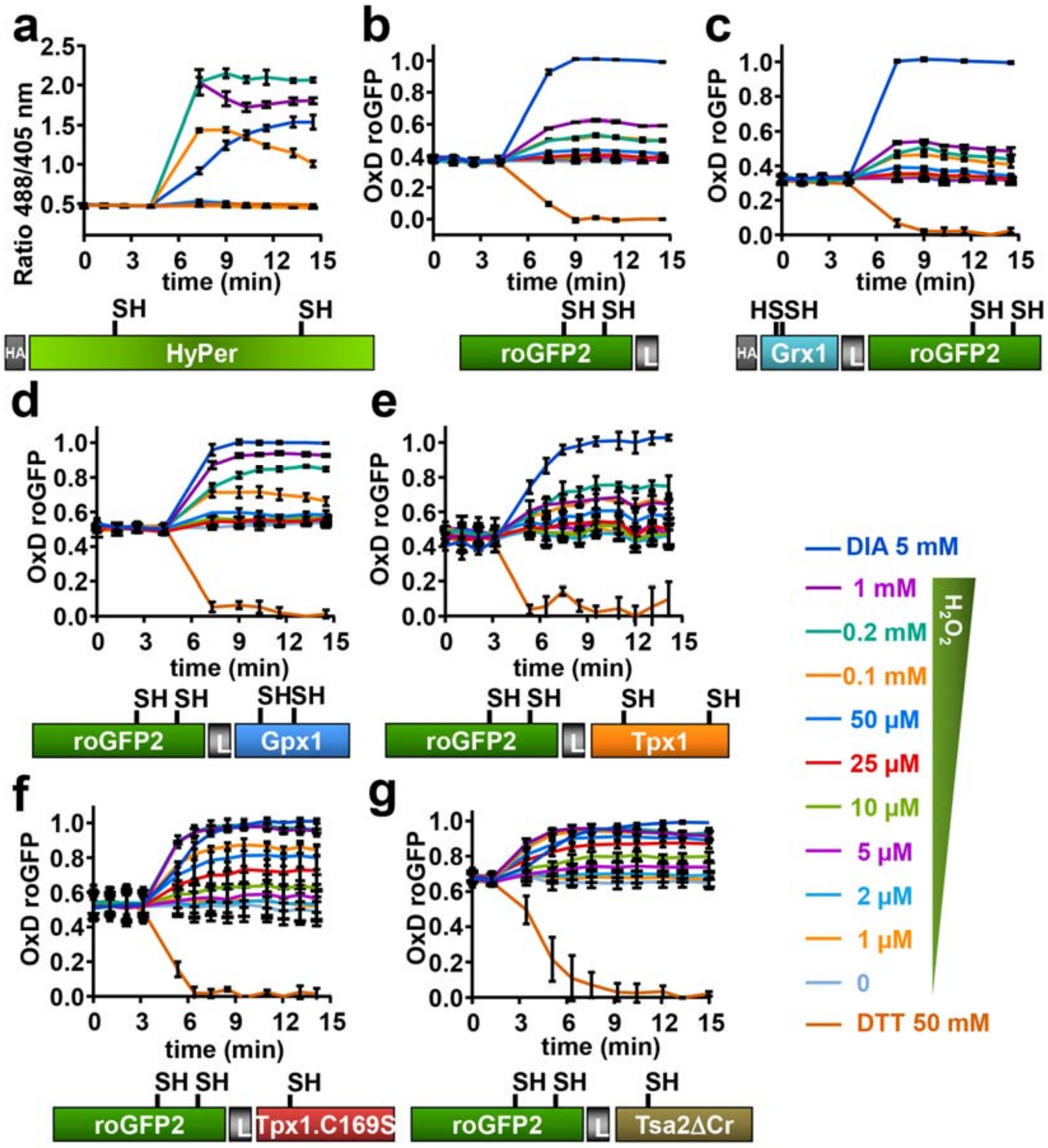

Supplementary Fig. 1 The response of HyPer (**a**), roGFP2 (**b**), Grx1-roGFP2 (**c**), roGFP2-Gpx1 (**d**), roGFP2-Tpx1 (**e**), roGFP2-Tpx1.C169S (**f**) and roGFP2-Tsa2ΔC_R (**g**) in wild-type cells treated with the indicated concentrations of H_2O_2, diamide and DTT. Data from 3 biological replicates with error bars (S.D.) are shown.

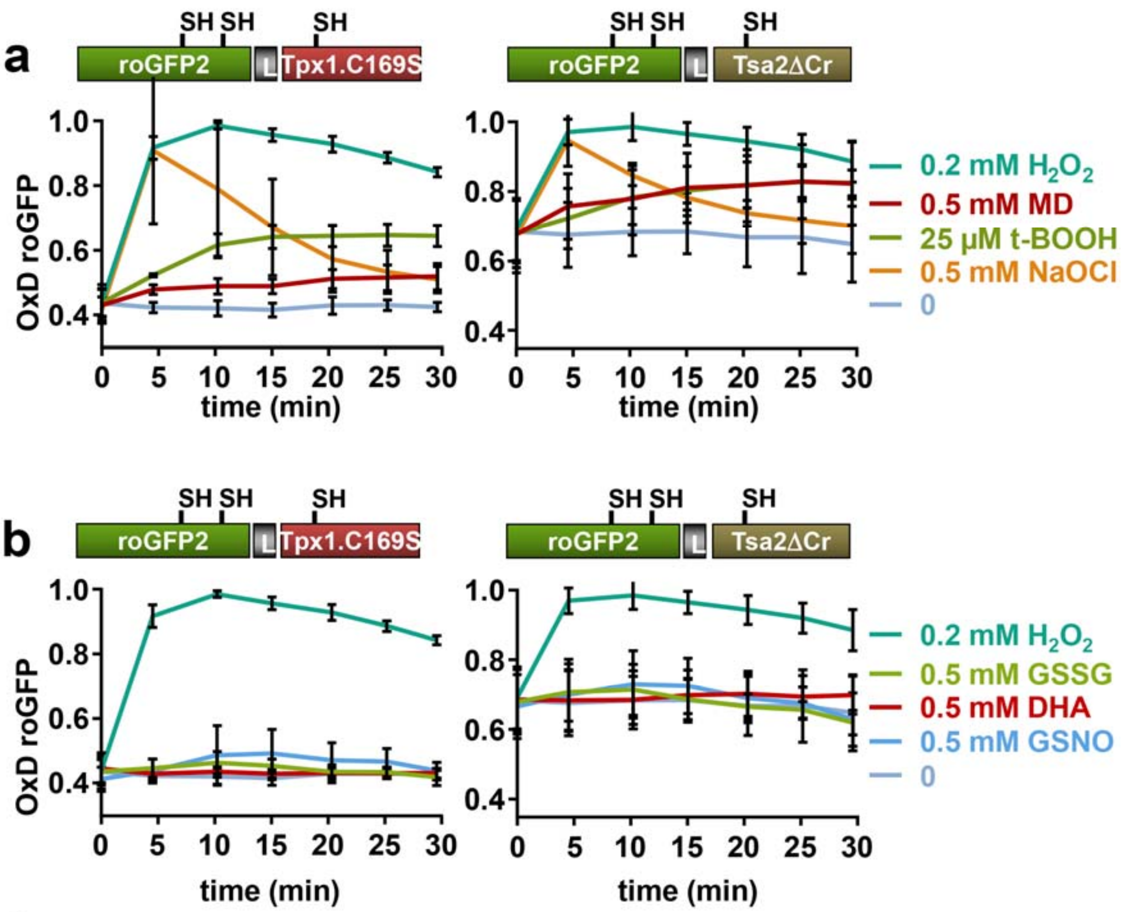

c

MSKGEELFTGVVPILVELDGDVNGHKFSVSGEGEGDATYGKLTLKFISTTGKLPVPWPTLVTTLTYGVQCFS
RYPDHMKQHDFFKSAMPEGYVQERTIFFKDDGNYKTRAEVKFEGDTLVNRIELKGIDFKEDGNILGHKLEYN
YNCHNVYIMADKQKNGIKVNFKIRHNIEDGSVQLADHYQQNTPIGDGPVLLPDNHYLSTCSALSKDPNEKRD
HMVLLEFVTAAGITLGMDELYK**TS**GGSGGGGSGGGGSGGGGSGGGGSGGGGSGG**PI**SLQIGKPAPDFKG
TAVVNGAFEEIKLADYKGKWVFLGFYPLDFTFVCPTEIVAFSEAASKFAERNAQVILTSTDSEYSHLAFINTPR
KEGGLGGINIPLLADPSHKVSRDYGVLIEDAGVAFRGLFLIDPKGVLRQITINDLPVGRSVDEALRLLDAFQFV
EEHGEVSPANWHKGSDTIDTKNPEKYFSKH

Supplementary Fig. 2 The *in vivo* response of roGFP2-Tpx1.C169S and roGFP2-Tsa2ΔC_R in wild-type cells treated with the indicated concentrations of tert-butyl hydroperoxide (t-BOOH), sodium hypochlorite (NaOCl) and menadione (MD) (**a**), and dehydroascorbic acid (DHA), S-nitrosoglutathione (GSNO) and oxidized GSH (GSSG) (**b**), at the indicated concentrations. Addition of H_2O_2 and media were used as controls in both panels. Data from 3 biological replicates with error bars (S.D.) are shown. **c** Protein sequence of the new H_2O_2 reporter, roGFP2-Tpx1.C169S. roGFP is indicated in green, the glycine-serine-rich linker region in grey and Tpx1.C169S in red. In black, two amino acids were introduced between the different protein domains due to the restriction enzyme-based cloning strategy. The Cys169-to-Ser mutation is underlined.

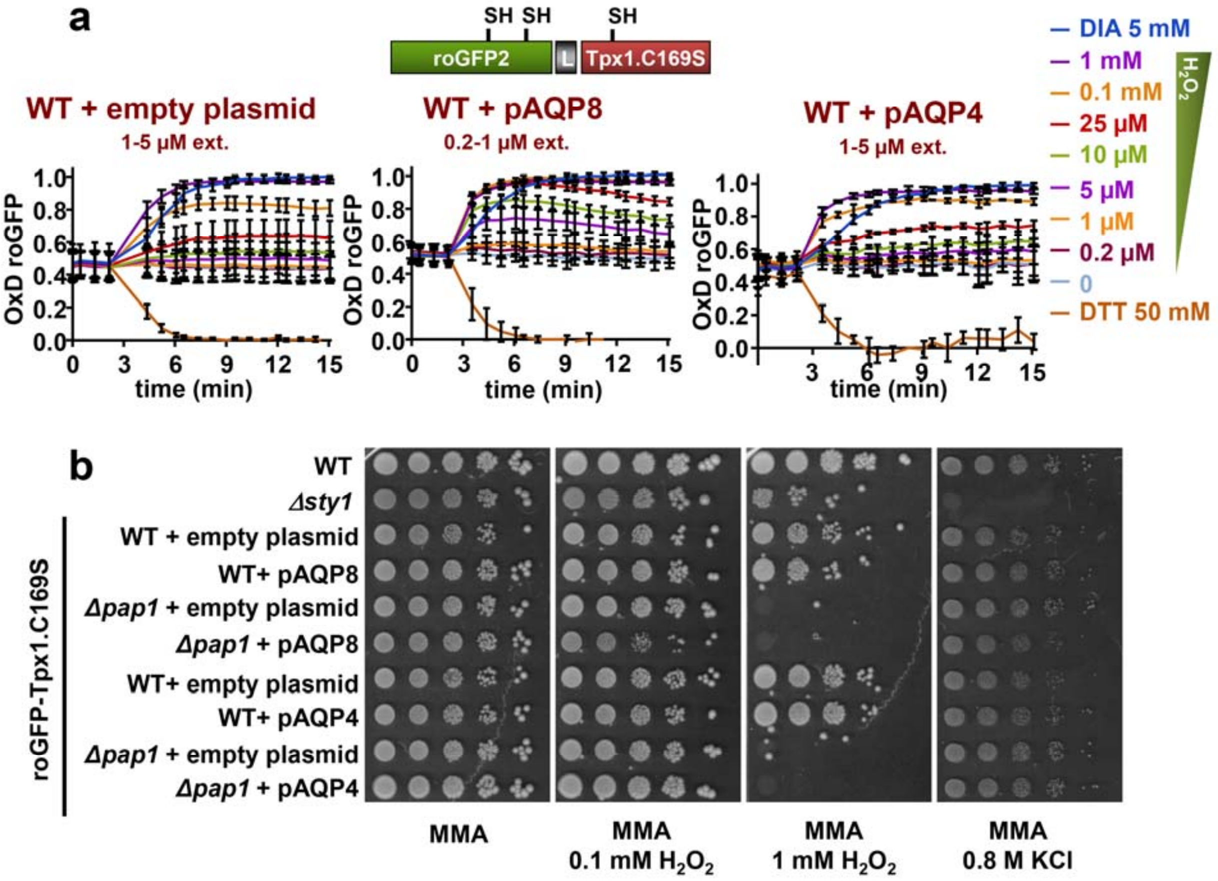

Supplementary Fig. 3 Oxidation of roGFP2-Tpx1.C169S upon addition of extracellular peroxides is exacerbated by expression of human AQP8, but not AQP4. **a** Wild-type strain PN513 was co-transformed with plasmid p407.C169S, coding for roGFP2-Tpx1.C169S, and an empty plasmid (left), plasmid p675 (center) or p682 (right) to trigger the constitutive expression of AQP8 or AQP4, respectively. H_2O_2 treatments were performed and oxidation of the reporter estimated as described in Fig. 1. Data from 3 biological with error bars (S.D.) are shown. **b** Expression of AQP8, but not of AQP4, in fission yeast decreases wild-type and *Δpap1* tolerance to peroxides. Strains PN513 (WT) or SG63 (*Δpap1*) were transformed with plasmid p407.C169S (coding for roGFP-Tpx1.C169S) and either an empty plasmid, p675 (coding for AQP8) or p682 (coding for AQP4). Serial dilutions of MM cultures of strains 972 (WT), AV18 (*Δsty1*), and the above transformants were spotted on MM agar plates containing or not the indicated concentrations of H_2O_2 or KCl, and grown for 3-4 days at 30°C.

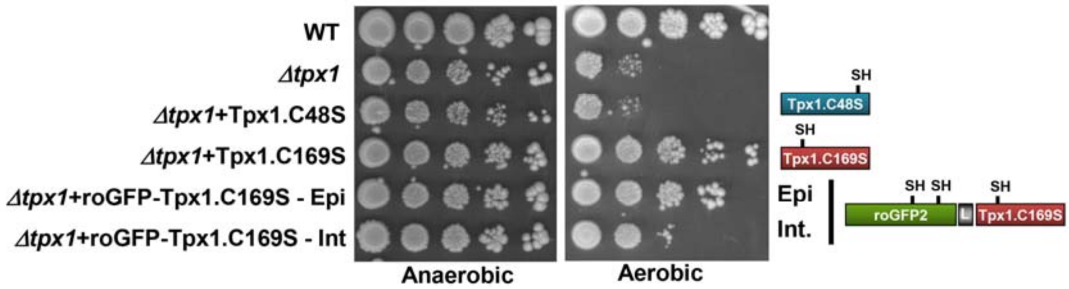

Supplementary Fig. 4 High concentrations of roGFP2-Tpx1.C169S can partially alleviate the aerobic growth defects of cells lacking Tpx1. Serial dilutions of MM cultures of strains 972 (WT), SG4 (*Δtpx1*), AV49.C48S (*Δtpx1*+Tpx1.C48S), AV49.C169S (*Δtpx1*+Tpx1.C169S), SG5 with plasmid p407.C169S (*Δtpx1*+roGFP-Tpx1.C169S - Epi) and MC167.C169S (*Δtpx1*+roGFP-Tpx1.C169S - Int) were spotted on MM agar plates and grown for 3-4 days at 30°C under anaerobic or aerobic conditions.

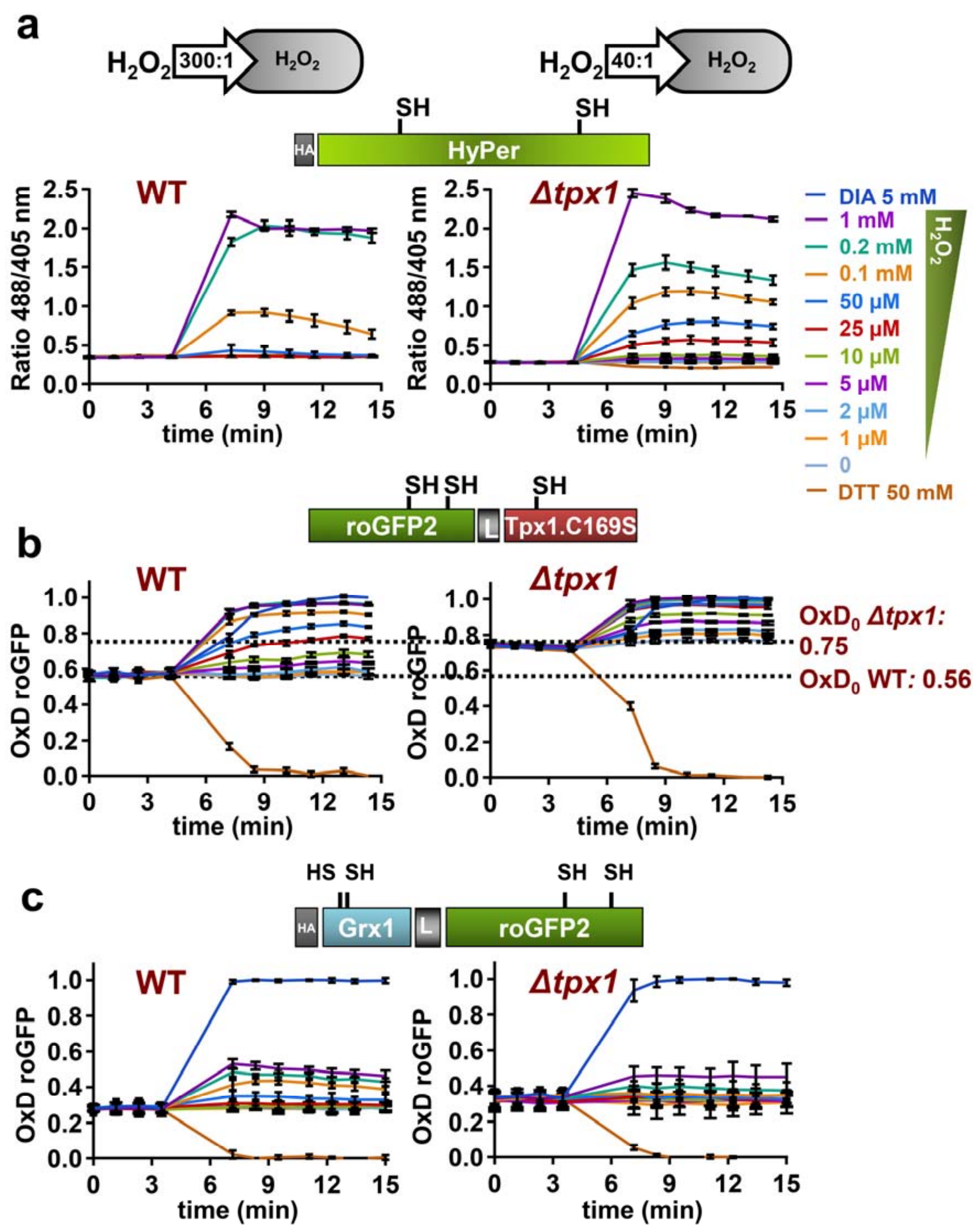

Supplementary Fig. 5 The response of HyPer (**a**), roGFP2-Tpx1.C169S (**b**) and Grx1-roGFP2 (**c**) in wild-type and *Δtpx1* cells treated with the indicated concentrations of H_2O_2, diamide and DTT. Data from 3 biological replicates with error bars (S.D.) are shown.

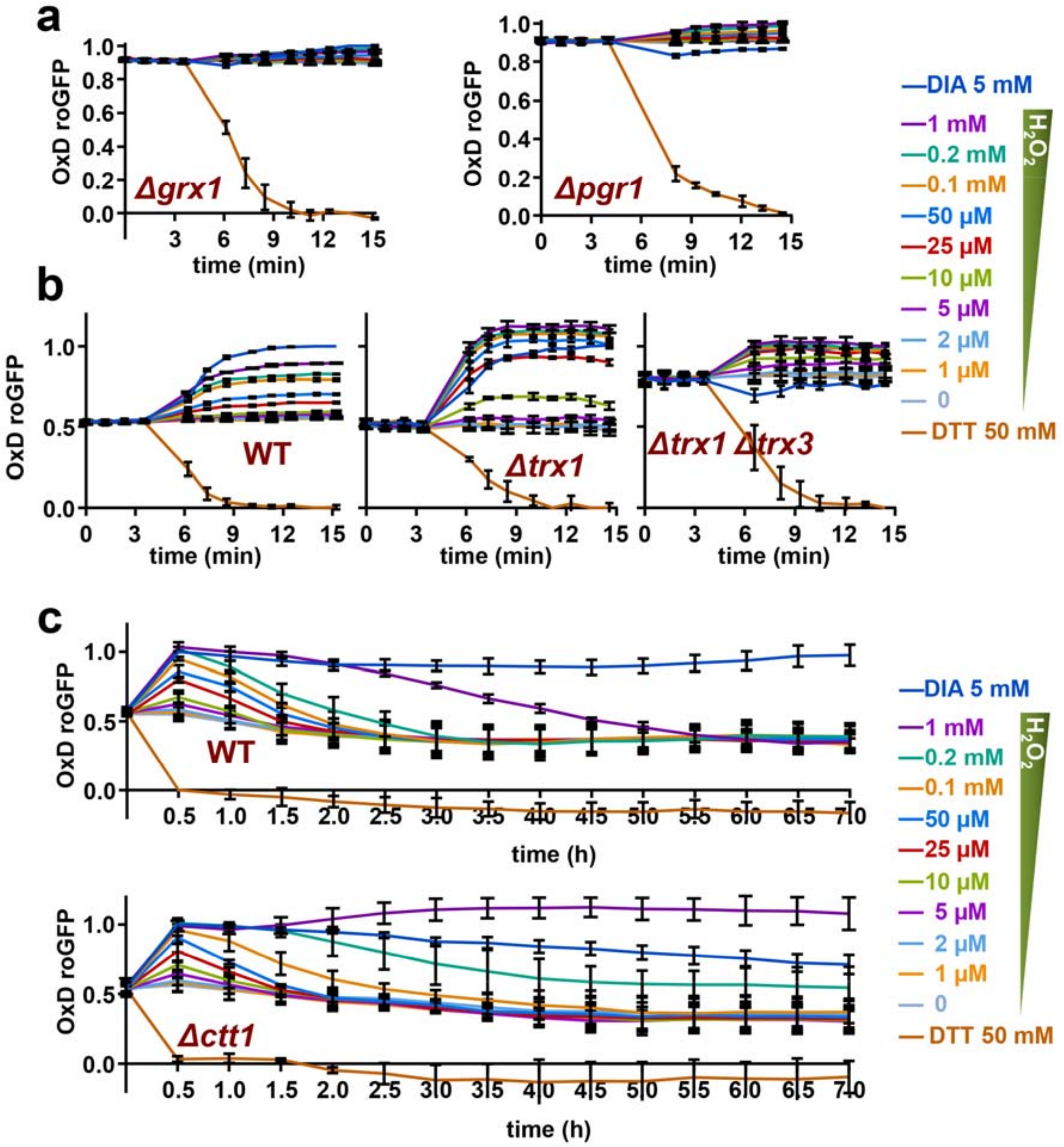

Supplementary Fig. 6 Response of roGFP2-Tpx1.C169S in wild-type (**b**,**c**), *Δgrx1* and *Δpgr1* (**a**), *Δtrx1* and *Δtrx1 Δtrx3* (**b**) and *Δctt1* (**c**) cells treated with the indicated concentrations of H_2O_2, diamide and DTT. Data from 3 biological replicates with error bars (S.D.) are shown.

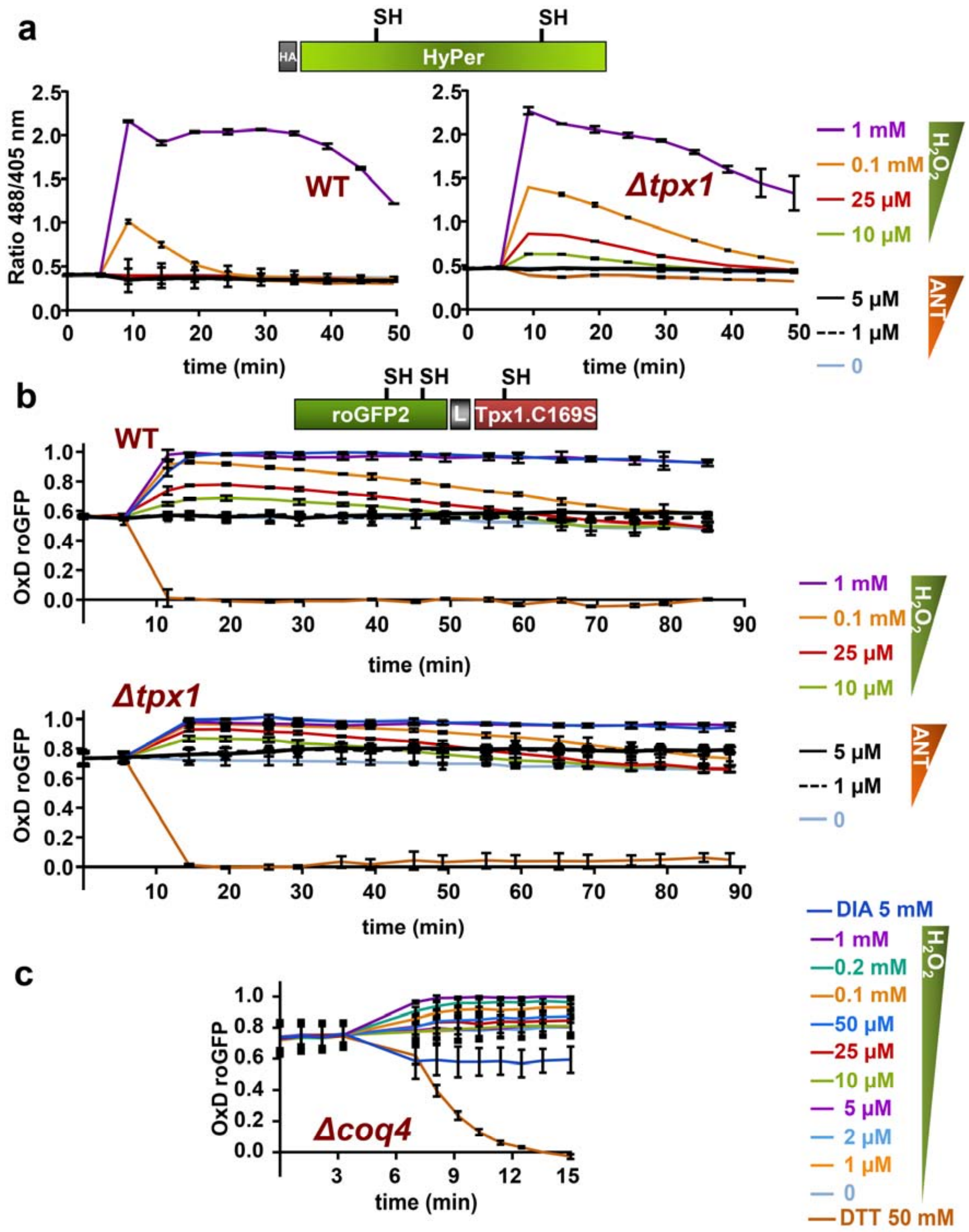

Supplementary Fig 7 Response of HyPer (**a**) and roGFP2-Tpx1.C169S (**b-c**) in wild-type (**a, b**), *Δtpx1* (**a, b**) and *Δcoq4* (**c**) cell cultures treated with the indicated concentrations of H_2O_2, diamide, DTT, or antimycin (ANT). **a** Data from 2 biological replicates with error bars (S.D.) are shown. **b** Data from 2 (WT) or 3 (*Δtpx1*) biological replicates with error bars (S.D.) are shown. **c,** Data from 3 biological replicates with error bars (S.D.) are shown.

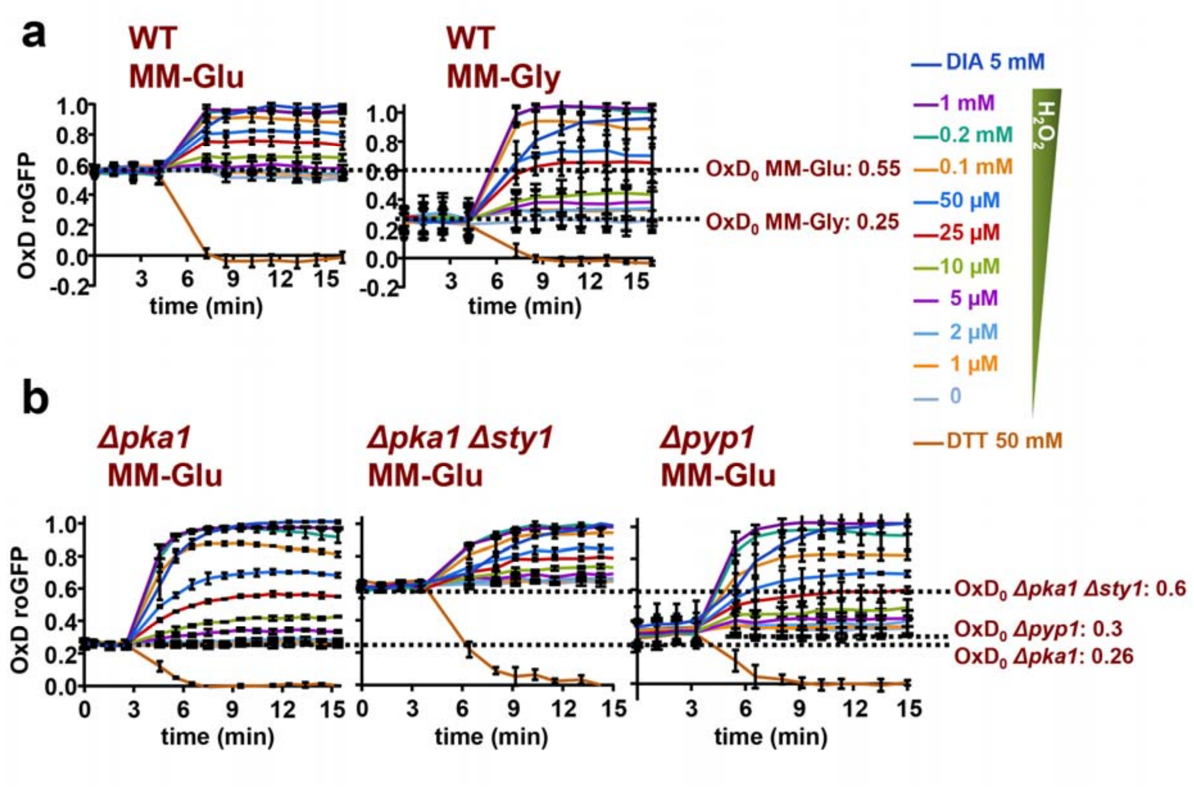

Supplementary Fig. 8 The response of roGFP2-Tpx1.C169S in wild-type (**a**), *Δpka1, Δpka1 Δsty1* and *Δpyp1* (**b**) cells treated with the indicated concentrations of H_2O_2, diamide and DTT, and grown in the indicated media. Data from 3 biological replicates with error bars (S.D.) are shown.

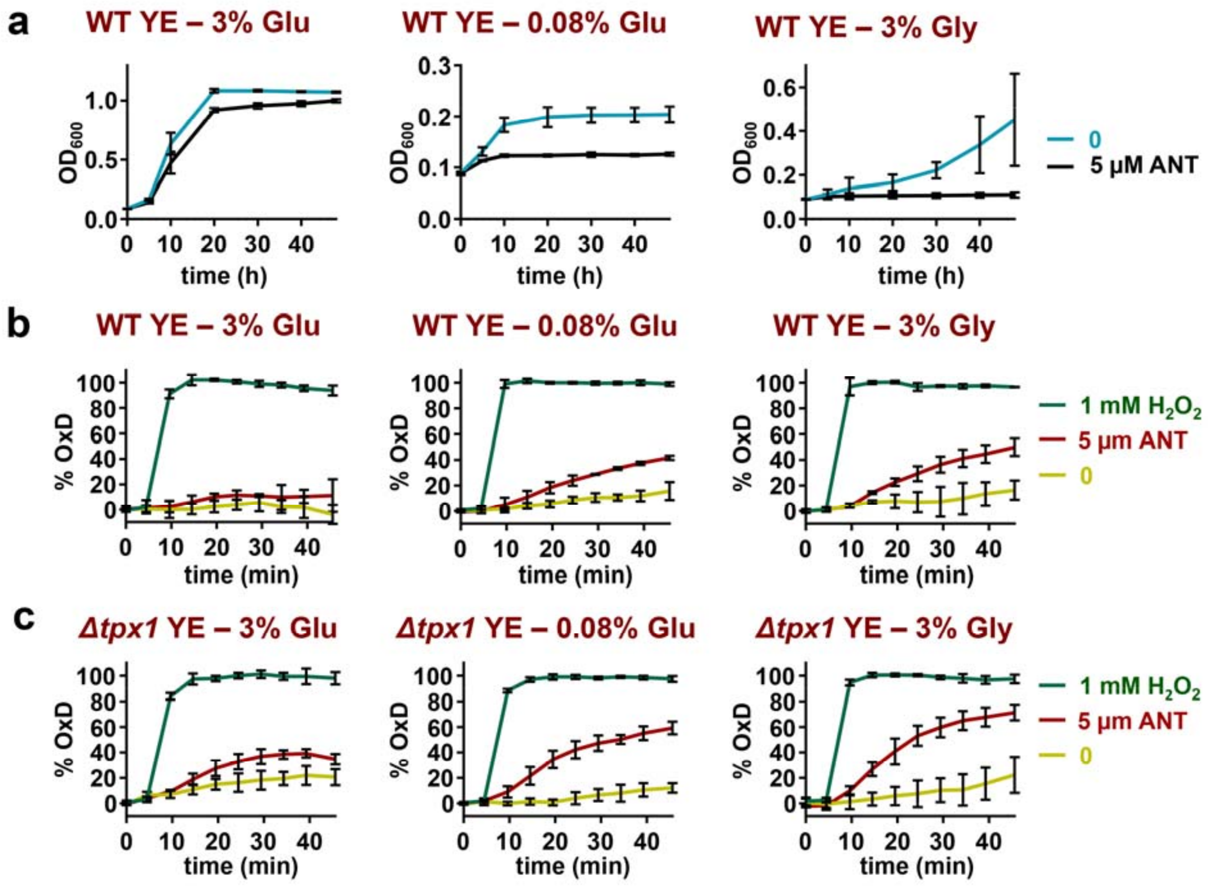

Supplementary Fig. 9 Antimycin A treatment triggers high levels of roGFP2-Tpx1.C169S oxidation in respiratory-prone culture media**. a** Growth of strain 972 in YE cultures with different carbon sources in the presence or absence of 5 µM ANT was monitored by recording OD_{600} for a period of ~50 h at 30°C. Data from 3 biological replicates with error bars (S.D.) is shown. **b, c** Strains HM123 (WT) (**b**) and SG5 (*Δtpx1*) (**c**) transformed with plasmid p407.C169S were grown in YE media containing 3% glucose (left panels), 0.08% glucose (center panels) or 3% glycerol (right panels). Cells were transferred to filtered MM, and probe oxidation upon ANT treatment (5 µM) was monitored for the time indicated. Probe oxidation in the absence of any oxidant or upon addition of 1 mM H_2O_2 is also shown for each strain and growth media condition. Data from 3 biological replicates with error bars (S.D.) are shown.

Supplementary Table 1. Strains used in this study

Strain	Genotype	Origin
972	h^-	[1]
AV18	h^- *sty1::kanMX6*	[2]
HM123	h^- *leu1-32*	Lab stock
PN513	h^- *ura4-D18 leu1-32*	Paul Nurse lab
364	h^+*ura4-D18*	Paul Nurse lab
SG63	h^+ *pap1::natMX6 ade6-M216 ura4-D18 leu1-32*	This work
SG4	h^+ *tpx1::natMX6*	[3]
SG5	h^+ *tpx1::natMX6 leu1-32*	[4]
AZ85	h^- *coq4::kanMX6*	[5]
Δ*coq4*	h^+ *coq4::kanMX6 ade6-M216 ura4-D18 leu1-32*	[6]
IC38	h^- *grx1::kanMX6*	[4]
SB36	h^+ *grx1::kanMX6 ura4-D18*	[7]
AD84	h^- *pgr1::natMX6*	[3]
AD88	h^+ *pgr1::natMX6 leu1-32*	This work
SG60	h^+ *trx1::natMX6*	This work
SG61	h^- *trx1::ura4 ura4-D18 leu1-32*	This work
SG260	h^- *trx1::kanMX6 trx3::natMX6*	This work
IC76	h^- *trx1::natMX6 trx3::kanMX6 ura4-D18 leu1-32*	[4]
EP198	h^+ *ctt1::natMX6*	[3]
EP160	h^- *ctt1::ura4 ura4-D18 leu1-32*	This work
EP16	h^- *sty1::ura4 leu1-32*	This work
EP193	h^- *atf1::natMX6 leu1-32*	[8]
ED1150	h^+ *cgs1:: ura4 leu1-32*	[9]
AZ74	h^- *pka1::kanMX6*	[10]
MC22	h^- *pka1::ura4 leu1-32*	[11]
AZ81	h^- *pka1::kanMX6 sty1::ura4*	This work
MC24	h^- *pka1::ura4 sty1::nat ura4-D18 leu1-32*	[11]
AZ103	h^- *pyp1::kanMX6*	[10]
AZ64	h^- *pyp1::kanMX6 leu1-32*	This work
AV49.C48S	h^+ *tpx1::kanMX6 tpx1'::tpx1.C48S::leu1 ade6-M216 ura4-D18*	[12]
AV49.C169S	h^+ *tpx1::kanMX6 tpx1'::tpx1.C169S::leu1 ade6-M216 ura4-D18*	[12]
MC167.C169S	h^+ *tpx1::natMX6 sty1'::roGFP2-L-tpx1.C169S::leu1*	This work

REFERENCES

1. Leupold, U. Genetical methods for *Schizosaccharomyces pombe*. *Methods Cell Physiol.* **4**, 169-177 (1970).
2. Zuin, A. et al. The glycolytic metabolite methylglyoxal activates Pap1 and Sty1 stress responses in Schizosaccharomyces pombe. *J Biol Chem* **280**, 36708-13 (2005).
3. Paulo, E. et al. A genetic approach to study H2O2 scavenging in fission yeast--distinct roles of peroxiredoxin and catalase. *Mol Microbiol* **92**, 246-57 (2014).
4. Calvo, I.A. et al. Dissection of a redox relay: H2O2-dependent activation of the transcription factor Pap1 through the peroxidatic Tpx1-thioredoxin cycle. *Cell Rep* **5**, 1413-24 (2013).
5. Shah, M. et al. A Transcript-Specific eIF3 Complex Mediates Global Translational Control of Energy Metabolism. *Cell Rep* **16**, 1891-902 (2016).
6. Kim, D.U. et al. Analysis of a genome-wide set of gene deletions in the fission yeast Schizosaccharomyces pombe. *Nat Biotechnol* **28**, 617-23 (2010).
7. Garcia-Santamarina, S., Boronat, S., Ayte, J. & Hidalgo, E. Methionine sulphoxide reductases revisited: free methionine as a primary target of H(2)O(2) stress in auxotrophic fission yeast. *Mol Microbiol* **90**, 1113-24 (2013).

8. Garcia, P. et al. Binding of the transcription factor Atf1 to promoters serves as a barrier to phase nucleosome arrays and avoid cryptic transcription. *Nucleic Acids Res* **42**, 10351-9 (2014).
9. Bonnet, C. et al. Identification and transcription control of fission yeast genes repressed by an ammonium starvation growth arrest. *Yeast* **16**, 23-33 (2000).
10. Zuin, A., Castellano-Esteve, D., Ayte, J. & Hidalgo, E. Living on the edge: stress and activation of stress responses promote lifespan extension. *Aging (Albany NY)* **2**, 231-7 (2010).
11. Zuin, A. et al. Lifespan extension by calorie restriction relies on the Sty1 MAP kinase stress pathway. *Embo J* **29**, 981-91 (2010).
12. Jara, M. et al. The peroxiredoxin Tpx1 is essential as a H2O2 scavenger during aerobic growth in fission yeast. *Mol Biol Cell* **18**, 2288-95 (2007).
