# Supplementary material for: Monitoring cytosolic H2O2 fluctuations arising from altered plasma membrane gradients or from mitochondrial activity
Source: Nat Commun. 2019 Oct 4;10:4526. doi: 10.1038/s41467-019-12475-0 (PMC6778086; doi:10.1038/s41467-019-12475-0)
Supplement: Supplementary file 3 — Reporting Summary [file 41467_2019_12475_MOESM3_ESM.pdf]

nature research

Corresponding author(s): Elena Hidalgo

Last updated by author(s): Sep 3, 2019

Reporting Summary

Nature Research wishes to improve the reproducibility of the work that we publish. This form provides structure for consistency and transparency in reporting. For further information on Nature Research policies, see Authors & Referees and the Editorial Policy Checklist.

Statistics

For all statistical analyses, confirm that the following items are present in the figure legend, table legend, main text, or Methods section.

n/a	Confirmed	
☐	☒	The exact sample size (*n*) for each experimental group/condition, given as a discrete number and unit of measurement
☐	☒	A statement on whether measurements were taken from distinct samples or whether the same sample was measured repeatedly
☐	☒	The statistical test(s) used AND whether they are one- or two-sided<br>*Only common tests should be described solely by name; describe more complex techniques in the Methods section.*
☒	☐	A description of all covariates tested
☒	☐	A description of any assumptions or corrections, such as tests of normality and adjustment for multiple comparisons
☐	☒	A full description of the statistical parameters including central tendency (e.g. means) or other basic estimates (e.g. regression coefficient) AND variation (e.g. standard deviation) or associated estimates of uncertainty (e.g. confidence intervals)
☒	☐	For null hypothesis testing, the test statistic (e.g. *F*, *t*, *r*) with confidence intervals, effect sizes, degrees of freedom and *P* value noted<br>*Give P values as exact values whenever suitable.*
☒	☐	For Bayesian analysis, information on the choice of priors and Markov chain Monte Carlo settings
☒	☐	For hierarchical and complex designs, identification of the appropriate level for tests and full reporting of outcomes
☒	☐	Estimates of effect sizes (e.g. Cohen's *d*, Pearson's *r*), indicating how they were calculated

Our web collection on statistics for biologists contains articles on many of the points above.

Software and code

Policy information about availability of computer code

Data collection	Fluorescence quantification directly on cell cultures using Fluorstar OMEGA (BMG Labtech)
Data analysis	Graphs were performed using PRISM (Graphpad). Figures were prepared using Power Point.

For manuscripts utilizing custom algorithms or software that are central to the research but not yet described in published literature, software must be made available to editors/reviewers. We strongly encourage code deposition in a community repository (e.g. GitHub). See the Nature Research guidelines for submitting code & software for further information.

Data

Policy information about availability of data

All manuscripts must include a data availability statement. This statement should provide the following information, where applicable:

- Accession codes, unique identifiers, or web links for publicly available datasets
- A list of figures that have associated raw data
- A description of any restrictions on data availability

All data generated or analyzed during this study are included in this article (and its supplementary figure files) and are available from the corresponding author on reasonable request. No accession codes, unique identifiers, or web links for publicly available datasets available.

Field-specific reporting

Please select the one below that is the best fit for your research. If you are not sure, read the appropriate sections before making your selection.

☒ Life sciences ☐ Behavioural & social sciences ☐ Ecological, evolutionary & environmental sciences

Life sciences study design

All studies must disclose on these points even when the disclosure is negative.

Sample size	A basis of n=3 was set as minimum sample size, unless indicated in some figure legends (n=2). 3 different yeast cultures were grown independently and measured in the same 96-well plate (biological replicates). Wild-type cells expressing the same reporter was always added in each 96-well plate, as a control.
Data exclusions	This study does not require data exclusions
Replication	For in vivo fluorescence probe oxidation quantification, one independent culture of the strain of interest was grown for each biological replicate. The exact n value is described in each figure legend, being 3 in most cases. In the case of spot assays, 3-to-4 independent experiments were performed, and all replicate experiments were successful.
Randomization	This study does not involve subjects that require randomization.
Blinding	This study does not involve procedures that require blinding.

Reporting for specific materials, systems and methods

We require information from authors about some types of materials, experimental systems and methods used in many studies. Here, indicate whether each material, system or method listed is relevant to your study. If you are not sure if a list item applies to your research, read the appropriate section before selecting a response.

Materials & experimental systems

n/a	Involved in the study
☒	☐ Antibodies
☒	☐ Eukaryotic cell lines
☒	☐ Palaeontology
☒	☐ Animals and other organisms
☒	☐ Human research participants
☒	☐ Clinical data

Methods

n/a	Involved in the study
☒	☐ ChIP-seq
☒	☐ Flow cytometry
☒	☐ MRI-based neuroimaging
